# Tendon stem cell-derived exosomes regulate inflammation and promote the high-quality healing of injured tendon

**DOI:** 10.1186/s13287-020-01918-x

**Published:** 2020-09-17

**Authors:** Mingzhao Zhang, Hengchen Liu, Qingbo Cui, Peilin Han, Shulong Yang, Manyu Shi, Tingting Zhang, Zenan Zhang, Zhaozhu Li

**Affiliations:** Department of Pediatric Surgery, The Second Affiliated Hospital of Harbin Medical University, No. 246, Xuefu Road, Nangang District, Harbin, 150001 China

**Keywords:** Exosomes, Tendon stem cells, Proliferation, Migration, Inflammation, Tendon healing

## Abstract

**Background:**

Tendon stem cells (TSCs) have been reported to hold promises for tendon repair and regeneration. However, less is known about the effects of exosomes derived from TSCs. Therefore, we aimed to clarify the healing effects of TSC-derived exosomes (TSC-Exos) on tendon injury.

**Methods:**

The Achilles tendons of Sprague-Dawley male rats were used for primary culture of TSCs and tenocytes, and exosomes were isolated from TSCs. The proliferation of tenocytes induced by TSC-Exos was analyzed using an EdU assay; cell migration was measured by cell scratch and transwell assays. We used western blot to analyze the role of the PI3K/AKT and MAPK/ERK1/2 signaling pathways. In vivo, Achilles tendon injury models were created in Sprague-Dawley rats. Rats (*n* = 54) were then randomly assigned to three groups: the TSC-Exos group, the GelMA group, and the control group. We used immunofluorescence to detect changes in the expression of inflammatory and apoptotic markers at 1 week after surgery. Histology and changes in expression of extracellular matrix (ECM)-related indices were assessed by hematoxylin-eosin (H&E) staining and immunohistochemistry at 2 and 8 weeks. The collagen fiber diameter of the healing tendon was analyzed at 8 weeks by transmission electron microscopy (TEM).

**Results:**

TSC-Exos were taken up by tenocytes, which promoted the proliferation and migration of cells in a dose-dependent manner; this process may depend on the activation of the PI3K/AKT and MAPK/ERK1/2 signaling pathways. At 1 week after surgery, we found that inflammation and apoptosis were significantly suppressed by TSC-Exos. At 2 and 8 weeks, tendons treated with TSC-Exos showed more continuous and regular arrangement in contrast to disorganized tendons in the GelMA and control groups, and TSC-Exos may help regulate ECM balance and inhibited scar formation. Further, at 8 weeks, the TSC-Exos group had a larger diameter of collagen compared to the control group.

**Conclusions:**

Our data suggest that TSC-Exos could promote high-quality healing of injured tendon, which may be a promising therapeutic approach for tendon injury.

## Introduction

Tendon injury is a common problem impacting patients’ work and lives and occurs most often during sports and recreational activities [1, 2]. Tendon healing is a long and complex process comprised of inflammation, proliferation, and remodeling. Because tendon tissue has minimal vascular supply and excessive loading, its ability to self-repair and regenerate after injury is poor [3]. Early, strong inflammatory reactions often lead to the formation of adhesions and scarring; scar formation, in turn, can weaken the tendon and increase the risk of re-tear [4]. Therefore, it is of particular importance that treatment of tendon injury focuses on anti-inflammation and inhibiting scar formation.

The study of tendon stem cells (TSCs), which are progenitor cells found in the tendon tissue, has received increased attention in recent years. TSCs have the potential of self-renewal and differentiation and have been widely used in tissue engineering and tendon injury healing. Growing studies have shown that the application of TSCs can effectively promote the repair and regeneration of injured tendons [5–8].

Exosomes are small (30–150 nm) membrane vesicles that contain a variety of proteins, mRNA, and miRNA, which are an important mediator for cell-cell communication and can be secreted by a variety of cells [9–11]. In recent years, many studies have shown that exosomes secreted by mesenchymal stem cells regulate inflammation [12, 13] and promote wound healing [11, 14, 15]; however, the involvement of TSC-derived exosomes (TSC-Exos) in response to tendon injury remains unknown.

Therefore, we hypothesized that TSC-Exos play an important role in modulating inflammation and promoting high-quality healing of tendon injuries. To test this hypothesis, exosomes were isolated from TSCs. In vitro, we investigated the effects of TSC-Exos on the proliferation and migration of tenocytes and their related mechanisms. In vivo, we studied the effects of TSC-Exos on inflammation, regeneration, and healing after tendon injury.

## Materials and methods

### Animals

A total of 62 adult male Sprague-Dawley rats were used in this study: 5 were used for TSC culture, 3 were used for tenocytes’ isolation, and the remaining 54 were used as the animal models per se. Rats were randomized and assigned into three groups (*n* = 18 rats per group). At 1, 2, and 8 weeks after surgery, rats in each group (*n* = 6) were sacrificed for corresponding experimental tasks. All experiments were approved by the Harbin Medical University Ethics Committee (No. Ky2018-135).

### Isolation and identification of TSCs

Isolation and identification of rat TSCs were performed as previously described [16]. Briefly, 0.3% pentobarbital sodium (Sigma, 30 mg/kg) was used for intraperitoneal anesthesia in rats. In sterile conditions, the tendon tissues were then removed, carefully dissected, cut into pieces, and digested in 3 mg/mL of type I collagenase (Sigma-Aldrich, St. Louis, MO, USA). After a 70-μm cell filter filtration, the suspension turned into a single cell suspension, which was then cultured in Dulbecco’s modified Eagle’s medium (Gibco, Invitrogen, NY, Invitrogen Corporation, Grand Island, USA) containing 10% fetal bovine serum (Biological Industries, Kibbutz Beit-Haemek, Israel) and 1% penicillin-streptomycin antibiotic mixture (Beyotime, Shanghai, China). Cells were subcultured at 80% confluence. Cells at passages three were incubated with fluorescein isothiocyanate-conjugated antibodies (anti-CD90, anti-CD105, anti-CD44, anti-CD11b, and anti-CD106) (Biolegend San Diego, CA, USA) through flow cytometry. The multilineage differentiation potential of TSCs was determined by inducing the differentiation of cells in passage 3 into osteocytes, adipocytes, and chondrocytes (all the differentiation media were from Cyagen).

### Isolation and culture of tenocytes

Rat tenocyte isolation and culture were performed as previously described by Shi and Wu [17, 18]. Briefly, the tendon tissue from rat was cut into small pieces and placed in six-well culture plates. Dulbecco’s Modified Eagle Medium containing 10% fetal bovine serum and 1% penicillin-streptomycin was added. After 3–4 days, cells began to emerge from the pieces. Cells in passage 3–5 were used in the following experiments.

### Isolation and identification of TSC-Exos

When TSCs reached 80% fusion, the medium was replaced with a medium containing 10% serum without exosomes. After 24 h, the culture medium was collected and centrifuged sequentially at 300×*g* for 10 min, 3000×*g* for 10 min, and then 10,000×*g* for 30 min to remove cellular debris. The supernatants were then ultracentrifuged at 100,000×g for 2 h to precipitate the exosomes, which were then resuspended in 200 μL of PBS. The total protein concentration of exosomes was quantified using the bicinchoninic acid assay (Beyotime, Shanghai, China). Morphology and quality of exosomes were examined by transmission electron microscopy (TEM), and particle size was measured using a ZetaView Analyzer (Particle Metrix, Germany). Western blot was used to detect exosome surface markers CD9, TSG101, and Hsp70. The above experiments were repeated three times.

### PKH26 labeling of exosomes

The generation of PKH26-labeled TSC-Exos was performed as previously described [15]. Briefly, 250 μL of exosomes diluted with PBS was added to 250 μL of DiluentC (Sigma-Aldrich, St. Louis, MO, USA). At the same time, 2 μL of PHK26 dye was added to 500 μL of DiluentC to make the final PHK26 concentration of 1 × 10^−6^ M. After that, the two solutions were mixed and incubated at room temperature for 3–5 min. The excess labeled dye was neutralized with 1 mL of serum and removed by centrifugation. The PHK26 labeled exosomes and tenocytes were co-cultured in a serum-free medium overnight. Then, cells were counterstained with 5 μg/mL Hoechst (UE, China) for 20 min and observed under a fluorescence microscope (Olympus, Japan).

### Tenocytes’ treatment

In this study, a total of more than 1.2 × 10^7^ tenocytes was used. To investigate the proliferation and migration of tenocytes treated with TSC-Exos, approximately 5 × 10^6^ tenocytes were divided into four treatment groups, receiving 0, 25, 50, or 100 μg/mL of allogeneic TSC-Exos. To further study the related mechanisms, cells were pretreated with a PI3K/AKT inhibitor, LY294002, and a MAPK/ERK1/2 inhibitor, PD98059 (50 nM, both from MedChemExpress, Monmouth Junction, NJ, USA), for 1 h before treatment with TSC-Exos. Cells were harvested 30 min after treatment for western blotting and after 24 h for Edu, scratch, and transwell assays.

### EdU assay

The proliferation of tenocytes was examined using the EdU Imaging Kit (UE, China) according to the manufacturer’s protocol. In brief, following treatment with different concentrations of TSC-Exos, a total of 1 × 10^4^ tenocytes per group were incubated with 50 μM EdU for 4 h before fixation with 4% paraformaldehyde, permeabilization with 0.5% Triton X-100, and staining with the EdU kit. The cell nuclei were stained with 5 μg/mL Hoechst (UE, China) for 20 min. Quantification of EdU-positive cells was manually performed under a fluorescence microscope (Olympus, Japan). The test was repeated three times.

### Migration analysis of tenocytes

The migration ability of tenocytes treated with TSC-Exos was measured by the scratch test and transwell assays. The cell scratch test was performed as previously described [16]. Simply, a total of 1 × 10^5^ tenocytes per group were seeded in six-well plates. When the cells grew to 80% fusion, the tip of a P200 pipette was used to make a straight line. The wells were washed with PBS three times to remove debris and detached cells. After that, a serum-free medium containing different concentrations of TSC-Exos was added to each well. At 0 and 24 h, the cell migration of each group was recorded using a microscope. The healing area/initial gap area was measured as the migration index using the ImageJ software.

The transwell experiment was performed according to the manufacturer’s recommendations. Simply, a total of 1 × 10^4^ tenocytes per group were planted on the upper chamber of the transwell (Corning Inc., NY, USA) with 100 μL of serum-free medium. After that, 500 μL of a medium containing 10% serum without exosomes and different concentrations of TSC-Exos were added to the lower layer. After culturing for 24 h, tenocytes on the permeable membrane of the upper chamber were fixed with anhydrous ethanol, stained by crystal violet, then washed three times and wiped to remove non-migrated cells. Finally, the migrated cells on the underside of the membrane were counted under a microscope. The above test was repeated three times.

### Protein extraction and western blotting

The specific experimental methods are similar to those previously described [4]. Briefly, 200 μg exosomes or 3 × 10^5^ TSC-Exos-treated tenocytes per group were lysed with RIPA buffer (Beyotime, Shanghai, China). The protein concentration of the lysate was estimated using the bicinchoninic acid assay (Beyotime, Shanghai, China). For each sample, 20 μg of total protein was used for polyacrylamide gel electrophoresis. After that, the protein was transferred to polyvinylidene difluoride (PVDF) membranes. The primary antibodies used in this study were Rabbit monoclonal anti-CD9 (1:2000; Abcam), anti-TSG101 (1:2000; Abcam), anti-Hsp70 (1:1000; Abcam), anti-AKT (1:1000; Cell Signaling Technology), anti-phospho-AKT (1:2000; Cell Signaling Technology), anti-ERK1/2 (1:1000; Cell Signaling Technology), and anti-phospho-ERK1/2 (1:2000; Cell Signaling Technology). Horseradish peroxidase-conjugated goat anti-rabbit antibody (1:5000; Boster, China) was used as the secondary antibody. The test was repeated three times. ImageJ software was used to analyze the gray value of the final protein band.

### Surgical procedure and treatment

**Fig. 1:**
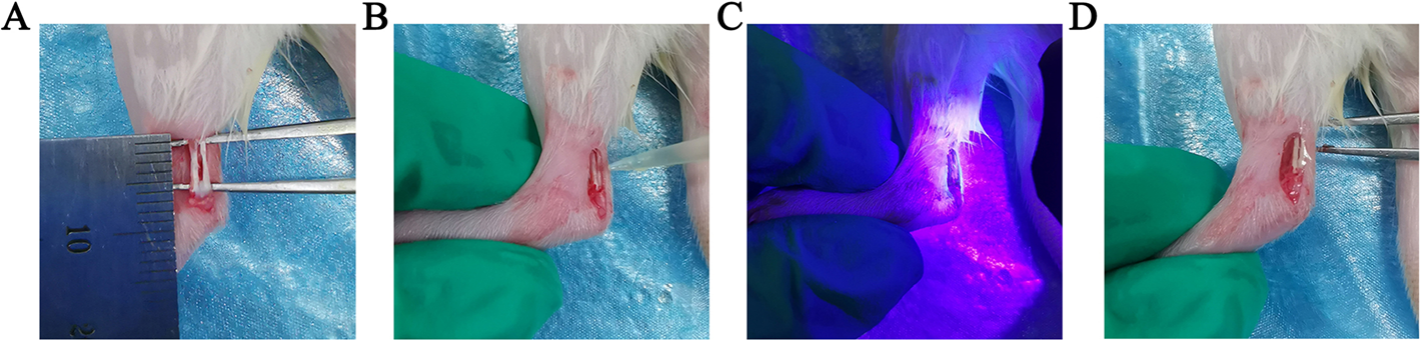
Achilles tendon injury model. **a** The central one third of the Achilles tendon was removed to create a tendon defect. **b** GelMA containing TSC-Exos was infused into the Achilles tendon defect. **c** Radiation with 405 nm light source for 10–20 s at a distance of 3 cm from the tendon defect. **d** Crosslinking to form the gel state

The specific surgical methods are similar to those employed in previous studies [17, 19]. Briefly, after anesthesia with 0.3% sodium pentobarbital (30 mg/kg, Sigma) in rats, a 2-cm-long incision was made in the right heel of rats to expose the Achilles tendon and remove a third of the central part of the tendon with a surgical blade, resulting in a central defect model of the Achilles tendon. Next, TSC-Exos were mixed in gelatin methacryloyl (GelMA, EFL-GM-60, 10%w/v) purchased from Suzhou Intelligent Manufacturing Research Institute (Suzhou, China). The mixture was placed in the Achilles tendon defect and converted to the gel state by irradiation for 10–20 s using a blue light source (3W, 405 nm) at a distance of 3 cm from the defect. The modeling process is shown in Fig. 1. In this study, we found that mixing 200 μg TSC-Exos into 30 μL GelMA was a reasonable choice; exceeding these doses would lead to loss of the mixture at the defect site, before it was converted to the gel state. Of note, to achieve proper gel formation, the appropriate GelMA concentration must be used, e.g., an excessive amount of TSC-Exos will influence the gel. Rats were randomly assigned to one of the following three groups (*n* = 18 each): the TSC-Exos group treated with 30 μL of GelMA containing 200 μg TSC-Exos, the GelMA group treated with 30 μL of GelMA alone, and the control group (untreated). A 5-0 non-absorbable surgical suture was used to close the skin incision, and buprenorphine (0.05 mg/kg) was administered subcutaneously for analgesia during the first 3 days postoperatively. After 1, 2, and 8 weeks, animals from each group (*n* = 6) were euthanized with an overdose of pentobarbital, and the repaired Achilles tendon was collected for immunostaining and TEM.

### Histology and immunostaining

The tissue specimens harvested at 1, 2, and 8 weeks after surgery were fixed in 4% paraformaldehyde solution, paraffin-embedded, and sectioned longitudinally at a thickness of 5 μm. For histology, sections at 2 and 8 weeks were processed for staining with hematoxylin-eosin (H&E) using standard protocols. For immunohistochemistry, the sections at 2 and 8 weeks were heated at 60 °C for 2 h. After deparaffinization and blocking with 3% H_2_O_2_, the sections were incubated in 5% normal goat serum for 15 min. After that, the sections were incubated at 4 °C overnight using the following antibodies: COL1a (rabbit polyclonal; 1:200; Abcam), COL3a1 (mouse monoclonal; 1:200; Abcam), MMP-9 (rabbit polyclonal; 1:200; Abcam), TIMP-1 (rabbit polyclonal; 1:200; Abcam), α-SMA (rabbit polyclonal; 1:200; Abcam), and fibronectin (mouse monoclonal; 1:200; Abcam). After washing with PBS, sections were incubated with secondary antibodies (1:500; Thermo Fisher) for 60 min. After washing again, the sections were stained with hematoxylin for 3 min. Finally, sections were dehydrated and mounted.

In order to reflect the diversity of experimental methods in the study, we used immunofluorescence staining to detect markers of inflammation and apoptosis, which were selected as previously described [17]. Briefly, after blocking with a blocking solution, the sections at 1 and 2 weeks were incubated at 4 °C overnight using the following antibodies: CD163 (rabbit monoclonal; 1:100; Abcam), C-C chemokine receptor type 7 (CCR7) (rabbit polyclonal; 1:200; Proteintech), interleukin (IL-10) (rat monoclonal 1:100; Abcam), IL-6 (mouse monoclonal; 1:100; Abcam), COX-2 (rat monoclonal; 1:100; Abcam), and cleaved caspase-3 (rabbit polyclonal; 1:100; Abcam). After washing with PBS, the sections were incubated with a secondary antibody (1:200; Proteintech) in the dark at room temperature for 60 min, and the nuclei were counterstained with 4′,6-diamidino-2-phenylindole (DAPI, Beyotime, Shanghai, China) for 10 min. All images were acquired with a BX53 microscope (Olympus, Japan) and analyzed by ImageJ software.

### Transmission electron microscopy imaging

For better observation of the ultrastructure of repaired tendons, we chose tissue samples collected 8 weeks after treatment, in theory better repaired, for TEM. Tissue samples were fixed, washed, dehydrated, mounted, and sputter-coated according to standard TEM methods described previously [20]. For each sample, we acquired about 8–10 images. The collagen fibers’ diameter was measured randomly by the measuring tool in TEM. Approximately 1000 collagen fibers in each group (*n* = 6) were measured to accurately reflect the distribution of collagen fiber diameters.

### Statistical analysis

All values are expressed as mean ± SD. Quantitative data across all groups were analyzed using one-way ANOVA followed by Tukey’s test. A *P* value of < 0.05 was considered to be statistically significant. Data concerning collagen fibers diameter was assessed for normality by histogram and Q-Q plots to choose parametric tests.

## Results

### Characterization of TSCs and TSC-Exos

**Fig. 2:**
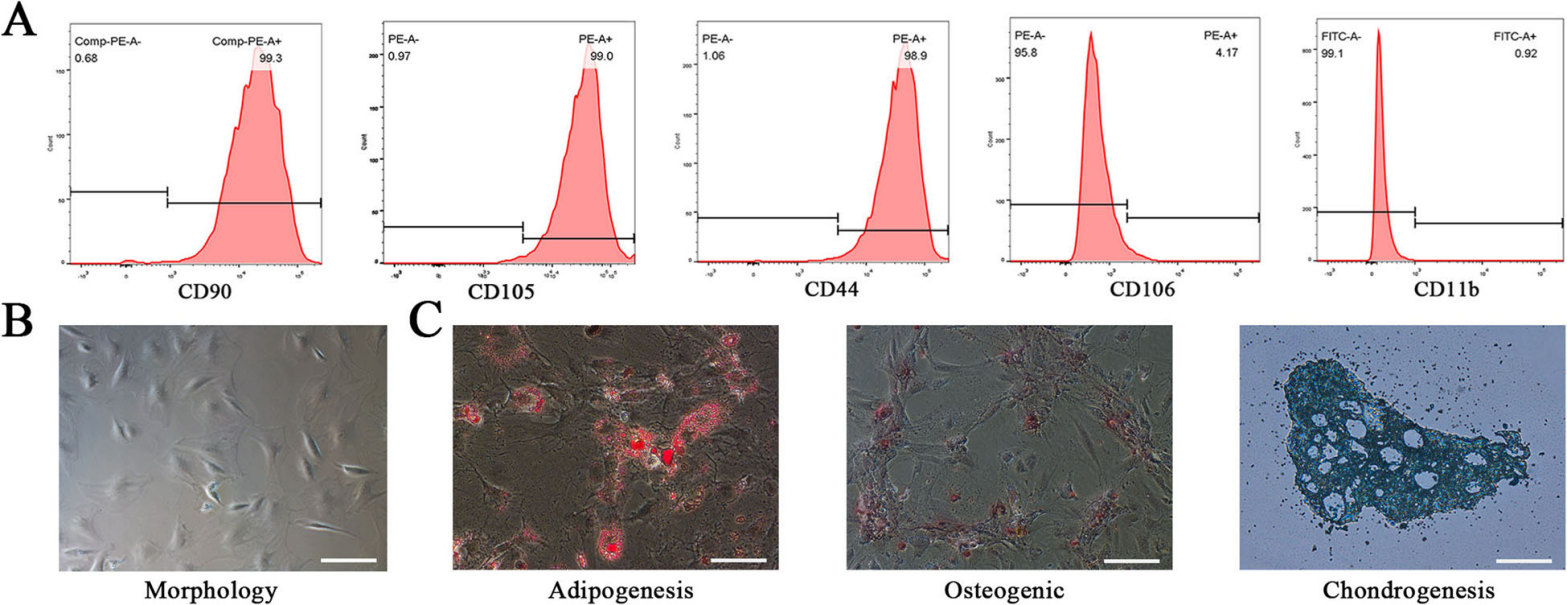
Characterization of TSCs. **a** Flow cytometry for detection of TSC surface markers. **b** Morphology of TSCs. **c** Adipogenic, osteogenic, and chondrogenic differentiation potential of TSCs. Bars, 100 μm

**Fig. 3:**
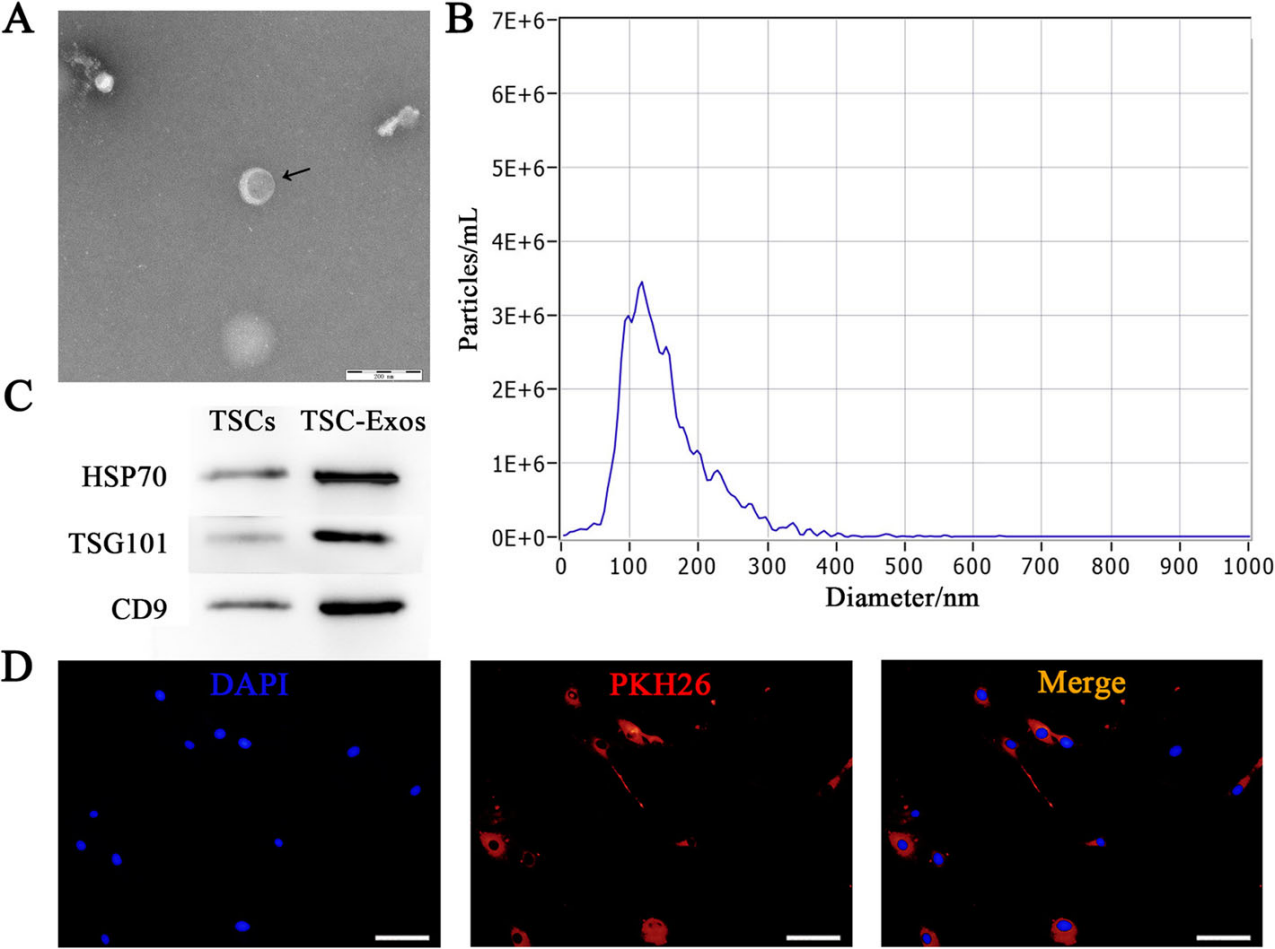
Characterization of TSC-Exos. **a** Morphology observed under transmission electron microscope. **b** Particle size distribution. **c** Western blot was used to detect exosomes surface markers. **d** Fluorescent microscopy analysis of PKH26-labeled TSC-Exos internalization by tenocytes. Nuclei were counterstained with DAPI. Bars, 100 μm

Results from flow cytometry showed the expression of positive markers CD90 (99.3%), CD105 (99.0%), and CD44 (98.5%), while negative markers CD106 and CD11b were 4.17% and 0.92%, respectively (Fig. 2a). TSCs showed a typical spindle-shaped morphology, with the potential of multilineage differentiation (Fig. 2b, c). Under TEM, TSC-Exos showed round vesicular structures (Fig. 3a), with an average size of 116 nm (Fig. 3b). Results of western blotting confirmed that exosomes expressed the specific markers CD9, TSG101, and HSP70 (Fig. 3c). Exosomes labeled with PKH26 red fluorescent dye were localized around the cell nucleus, indicating internalization of TSC-Exos by tendon cells (Fig. 3d). All these features were consistent with the previous studies.

### Cell proliferation and migration

**Fig. 4:**
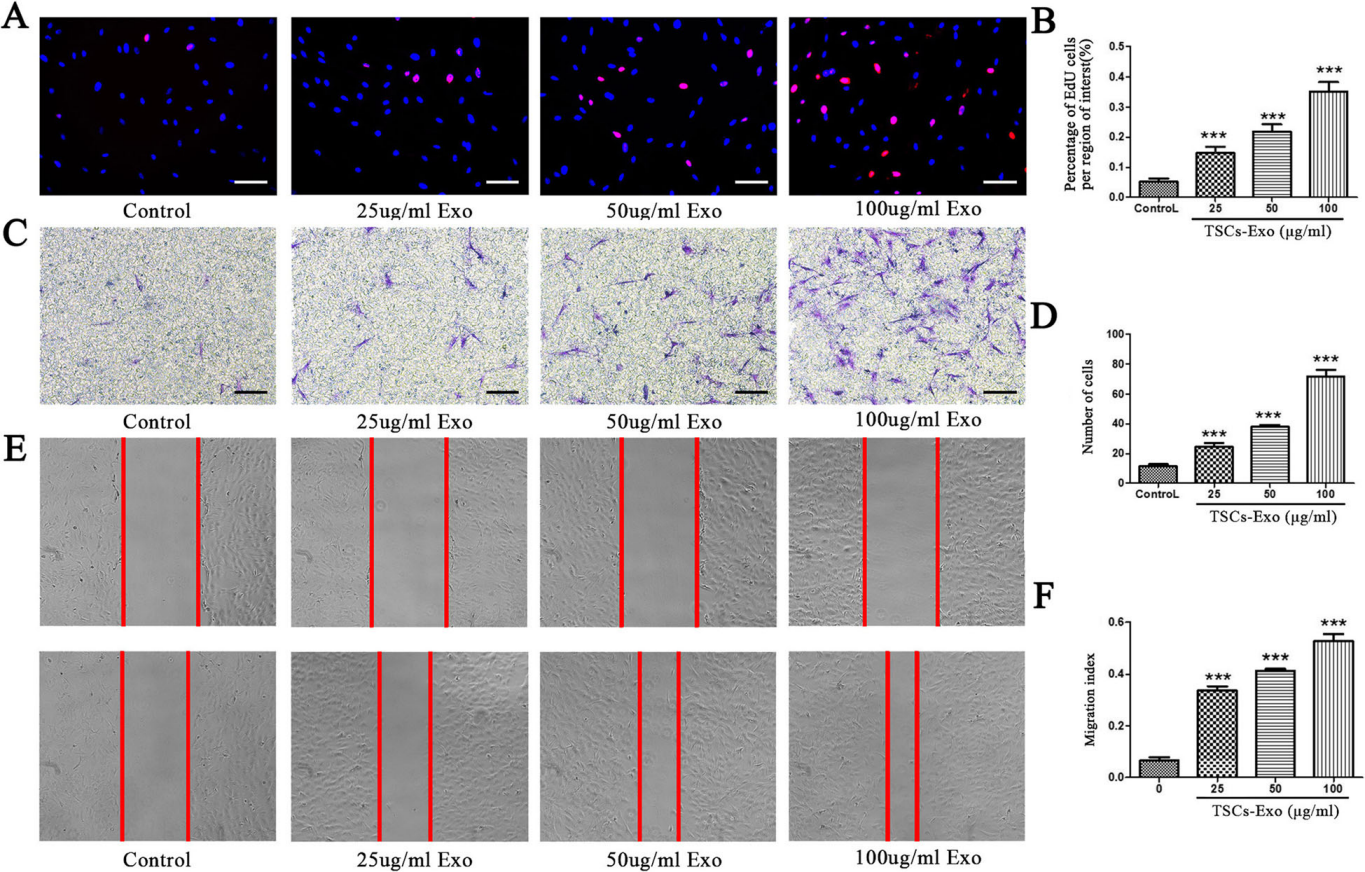
TSC-Exos significantly promote the proliferation and migration of tenocytes in a dose-dependent manner. **a, b** Effect of different concentrations of TSC-Exos on the proliferation of cells by EdU assays. The migration ability of tenocytes treated with TSCs-Exo, measured by **c**, **d** Transwell and **e**, **f** scratch test assays. Bars, 100 μm. Data are represented as mean ± SD. *, vs control group; *n* = 3. ***P < 0.0001

To measure the effect of TSC-Exos on tenocytes in detail, we first tested their effect on proliferation capability of tenocytes. Indeed, exosomes significantly promoted cell proliferation in a dose-dependent manner (Fig. 4a, b). Results from the transwell assay demonstrated that migration ability of tenocytes increases gradually for 24 h after treatment with different concentrations of exosomes (Fig. 4c, d). Similarly, the scratch test also showed the same result (Fig. 4e, f).

### Regulatory function of PI3K/AKT and MAPK/ERK1/2 signaling in tenocytes proliferation and migration

**Fig. 5:**
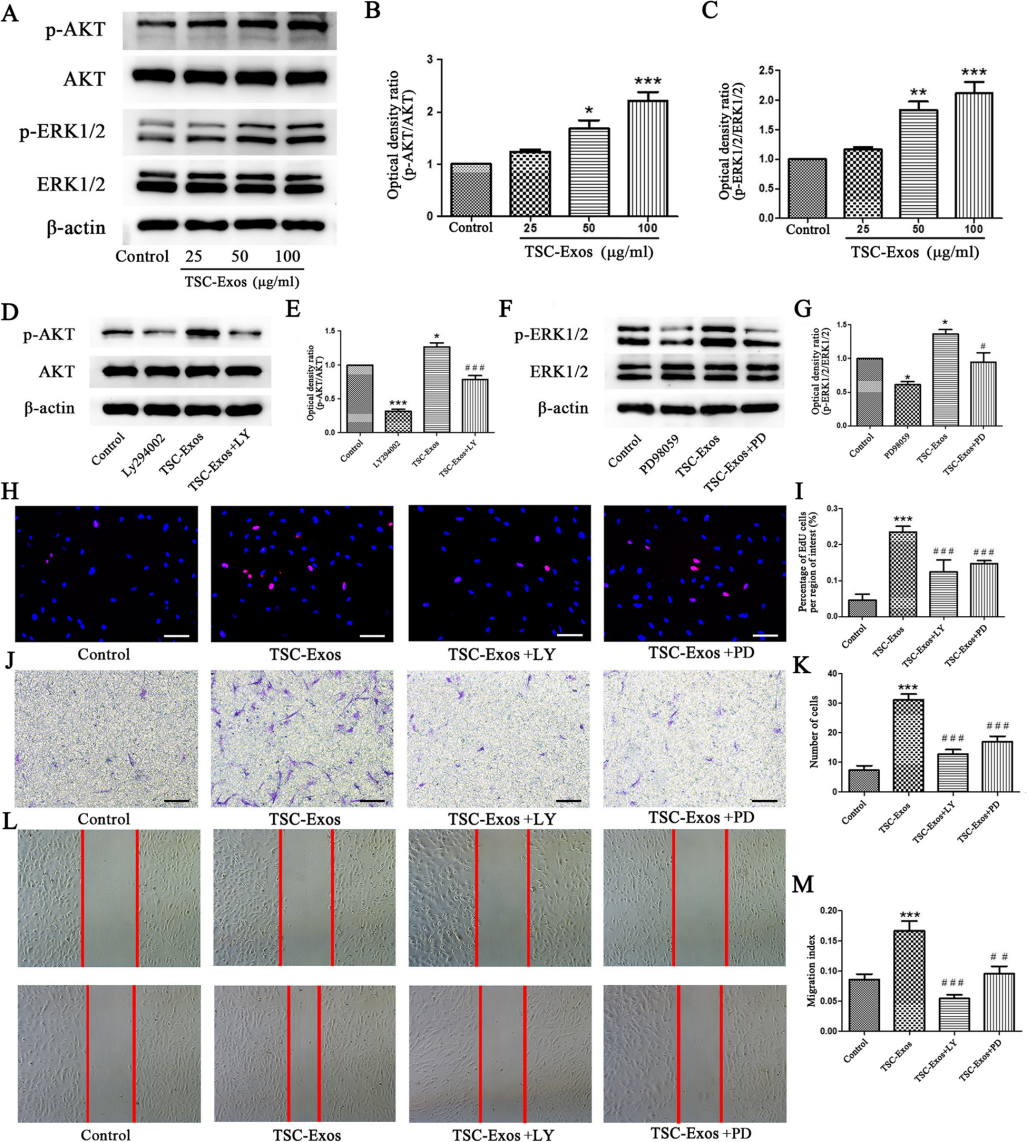
TSC-Exos promote the proliferation and migration of cells via the PI3K/AKT and MAPK/ERK1/2 signaling. **a–c** Western-blot analysis of protein levels of pAkt and pERK1/2 induced by different concentrations of TSC-Exos. **d**–**g** LY294002 and PD98059 inhibit the activation of PI3K/AKT and MAPK/ERK1/2 induced by TSC-Exos, respectively. **h**, **i** EdU assay showed that TSC-Exos-mediated tenocyte proliferation was suppressed by inhibitors LY294002 and PD98059. **j**, **k** Transwell and **l**, **m** scratch assays showed that TSC-Exos enhanced tenocyte migration at 24 h, but this effect was significantly reduced by inhibitors LY294002 and PD98059. Bars, 100 μm; data are represented as mean ± SD. *, vs control; #, vs TSCs-Exo, *n* = 3. *P < 0.05, **P < 0.01, ***P < 0.0001, ^#^*P* < 0.05, ^##^*P* < 0.01, ^###^*P* < 0.0001

The PI3K/AKT and MAPK/ERK1/2 signaling pathways play an important role in increasing cell proliferation and migration as well as facilitating wound healing. Therefore, we used western blot to evaluate the activity of these pathways. Results showed that TSC-Exos significantly increase protein levels of p-AKT and p-ERK1/2 in tenocytes in a dose-dependent manner (Fig. 5a–c), suggesting that regulation of TSC-Exos-dependent proliferation and migration may occur through PI3K/AKT and MAPK/ERK1/2 signals. To study the regulatory effect of these two signaling pathways on tenocytes further, we pretreated tenocytes with signal inhibitors, LY294002 and PD98059, before treatment with TSCs-Exos. The results showed that TSC-Exos-induced increases in phosphorylation of AKT and ERK1/2 were significantly inhibited by these signal inhibitors (Fig. 5d–g). Functionally, after inhibiting PI3K/AKT and MAPK/ERK1/2 signals, results from Edu assay showed that the effect of TSC-Exos on the proliferation of tenocytes was significantly weakened (Fig. 5h, i). Similarly, TSC-Exos promoted migration of tenocytes, but this promotion was also significantly suppressed by these two signal inhibitors (Fig. 5j–m).

### Effect of TSCs-Exos on the early inflammatory response

**Fig. 6:**
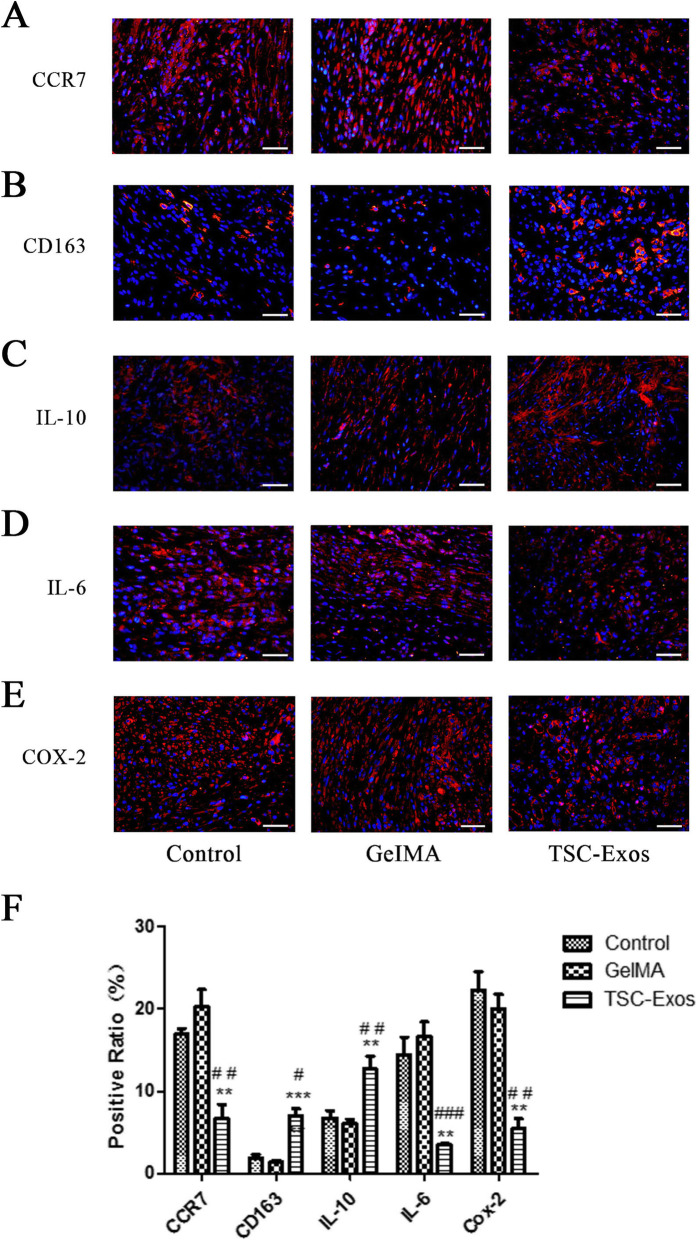
Impact of TSC-Exos on tendon inflammation. The expression of **a** CCR7+ cells, **b** CD163+ cells, **c** IL-10+ cells, **d** IL-6+cells, and **e** COX-2+ cells evaluated at 1-week post-surgery by immunofluorescence assay. **f** Positive cell ratio of inflammation-related factors at 1 week (*n* = 6). Bars, 50 μm. Data are represented as mean ± SD. *, vs control; #, vs GelMA group. **P < 0.01, ***P < 0.0001, ^#^*P* < 0.05, ^##^*P* < 0.01, ^###^*P* < 0.0001

We next studied the role of TSC-Exos in regulating the early inflammatory response in vivo. Expression of CCR7, a marker for type M1 pro-inflammatory macrophages, reduced significantly at 1 week in the TSC-Exos group compared to those in the other groups (Fig. 6a). By contrast, levels of CD 163 (a marker of type M2 anti-inflammatory macrophages) were significantly increased (Fig. 6b). Accordingly, the expression of IL-10 (M2 macrophage stimulating factor) was higher in the TSC-Exos group than that in the GelMA and control groups (Fig. 6c), while IL-6 (M1 macrophage stimulating factor) was significantly decreased (Fig. 6d). Furthermore, Cox-2, another a pro-inflammatory factor, decreased significantly in the treatment group (Fig. 6e). These results were confirmed quantitatively, with a significantly higher density of CD163+ and IL-10+ cells and fewer CCR7+, IL-6+, and Cox-2+ cells in the TSC-Exos group than in the other groups (Fig. 6f).

### Effect of TSC-Exos on cell apoptosis

**Fig. 7:**
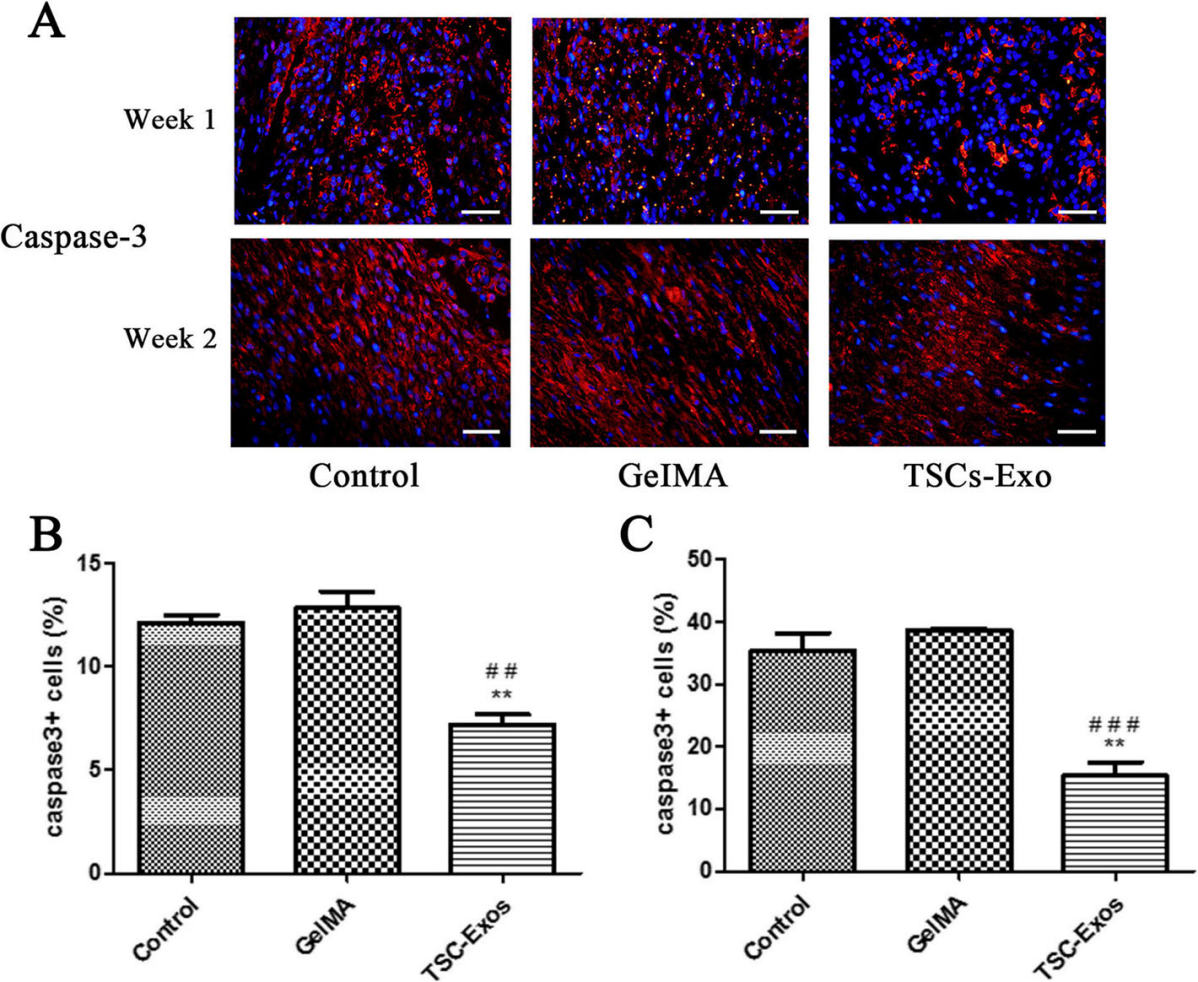
Impact of TSC-Exos on tendon cell apoptosis . **a** Cellular expression of caspase3+ at 1 and 2 weeks after surgery evaluated by immunofluorescence assay. **b** Ratio of caspase3+ cells at 1 week (*n* = 6). **c** Ratio of caspase3+ cells at 2 weeks (*n* = 6). Bars, 50 μm. Data are represented as mean ± SD. *, vs control; #, vs GelMA group. **P < 0.01, ^##^*P* < 0.01, ^###^*P* < 0.0001

Activation of M1 macrophages is closely related to the extent of apoptosis seen after tendon injury. Therefore, we tested whether TSC-Exos could block apoptotic cell death during tendon repair. Immunofluorescence results showed that the expression of cleaved caspase-3 (a marker of apoptotic cells) per group increased more than twice in week 2 compared to week 1. However, each week’s expression of cleaved caspase 3 in the TSCs-Exos group was significantly decreased compared to that in the GelMA and control groups (Fig. 7a–c).

### TSC-Exos improves tendon healing and regulates extracellular matrix formation

**Fig. 8:**
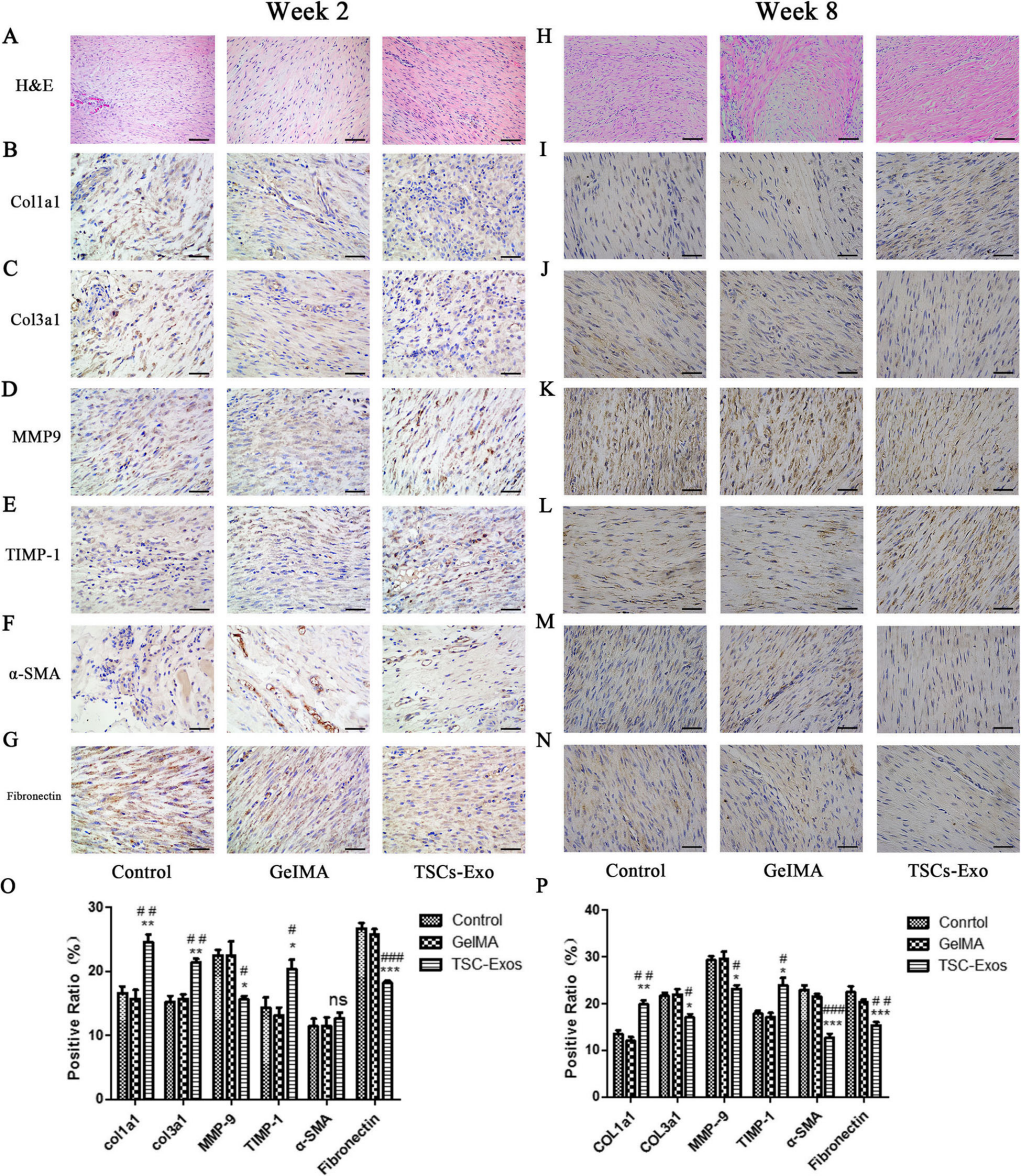
Impact of TSC-Exos on Histology and tendon matrix-related factors. **a**, **h** The HE staining of window defect on the Achilles tendon at 2 and 8 weeks post-surgery. Expression of **b** COL1a1, **c** COL3a1, **d** MMP-9, **e** TIMP-1, **f** α-SMA, and **g** fibronectin evaluated at 2 weeks by immunohistochemistry assay. **i**–**n** Expression of the above extracellular matrix-related factors after TSC-Exos treatment at 8 weeks. **o** Quantitative analysis of tendon matrix-related factors at 2 weeks. **p** Quantitative analysis of tendon matrix-related factors at 8 weeks. Bars, 50 μm. Data are represented as mean ± SD. *, vs control; #, vs GelMA group. *P < 0.05, **P < 0.01, ***P < 0.0001, ^#^*P* < 0.05, ^##^*P* < 0.01, ^###^*P* < 0.0001

H&E staining revealed that tendon in TSC-Exos treatment group showed more continuous and regular arrangement in contrast to disorganized tendon in the GelMA and control groups (Fig. 8a, h). We also studied the effect of TSC-Exos on tendon matrix-related factors, COL1a1, COL3a1, matrix metalloproteinase-9 (MMP-9), and tissue inhibitor of matrix metalloproteinase (TIMP-1). At 2 weeks, the expression of COL1a1, COL3a1, and TIMP-1 in TSC-Exos treatment group was upregulated (Fig. 8b, c, e) and that of MMP-9 was downregulated (Fig. 8d) compared with that in the control group and GelMA group. Furthermore, we also found that the relative level of COl1a1 in the TSC-Exos group was higher than that of COL3a1 (Fig. 8q). At 8 weeks, COL1a1 and TIMP-1 levels in the TSC-Exos treatment group remained significantly increased (Fig. 8i, l) and the expression of MMP-9 was decreased (Fig. 8k). By contrast, COl3a1 showed a significant downward trend (Fig. 8j).

α-SMA, a myofibroblast marker, showed a downward trend in TSC-Exos group at 8 weeks (Fig. 8m). However, there was no statistically significant difference in α-SMA levels observed between treatment groups at 2 weeks (Fig. 8f). The presence of fibronectin correlated with the formation of scar after tendon injury. At 2 and 8 weeks, the expression of fibronectin in TSC-Exos group significantly decreased (Fig. 8g, n). The results of all quantitative analyses are shown (Fig. 8q, p).

### Effect of TSC-Exos on collagen fiber diameter

**Fig. 9:**
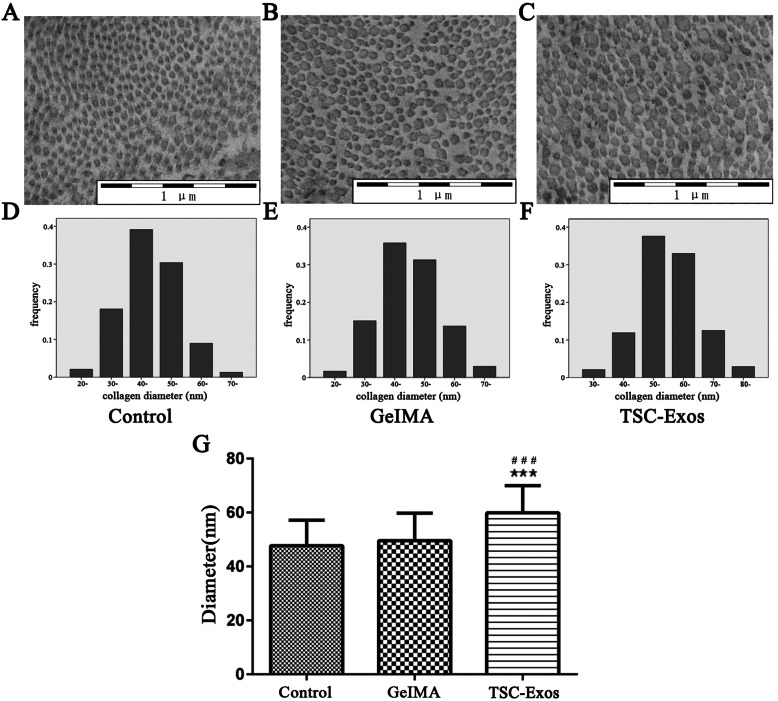
Ultrastructure observations of the repaired tendons at 8 weeks after surgery. Histogram and distribution of collagen fibril diameters in the control (**a, d**), GelMA (**b, e**), and TSC-Exos groups (**c, f**). **g** Quantitative analysis of collagen fiber diameter in each group (1000 fibers). Data are represented as mean ± SD. *, vs control; #, vs GelMA group. (*n* = 6). ***P < 0.0001, ^###^*P* < 0.0001

The ultrastructure of collagen fibers in the healing tendon tissue is shown in Fig. 9. Collagen fiber diameter in the TSC-Exos group (Fig. 9a–c) was found to be larger than that in the control and GelMA group. Figure 9d–f shows the collagen fiber diameter distribution in the treatment and control groups. The distribution of collagen fiber diameter size in each group was not uniform; diameter of collagen fibers in the GelMA and control groups were mainly within the range of 40–60 nm, while the TSC-Exos group had a lower percentage of smaller diameters that were within the range of 50–70 nm. Quantitative analysis of collagen fiber diameters of each group is shown in Fig. 9.

## Discussion

Repair following tendon injury is required to undergo three stages: inflammation, proliferation, and remodeling. Early, strong inflammatory responses can cause scar formation in the later stages of tendon healing [21]. After scar healing, tendons display changes in histology, biochemistry, and biomechanical properties, resulting in the inability to return to normal tendon strength and elasticity, and are likely to break again upon further injury [22]. Therefore, it is of great importance to control inflammation after tendon injury in order to promote high-quality healing. A growing number of studies have shown that mesenchymal stem cell-derived exosomes could inhibit the early inflammatory response in tendon injury and promote tissue healing [23–25]. However, the exact mechanisms by which TSC-Exos influence the healing of tendon injury remain unknown.

Proliferation and migration are indispensable processes in the repair of tendon injury [26]. In vitro, we first investigated the effects of TSC-Exos on the proliferation and migration of tenocytes. We found that TSC-Exos could promote the proliferation and migration of tenocytes in a dose-dependent manner. Exosomes from other cell sources show similar results, as numerous studies have shown that exosomes derived from mesenchymal stem cells can promote cell proliferation and migration, thus promoting the healing of tissue injury [14, 27]. It is well known that the PI3K/AKT and MAPK/ERK1/2 signaling pathways participate in the process of proliferation and migration of various types of cells [16]. Growing studies have shown exosomes could activate phosphorylation of survival pathways, especially of the MAPK/ERK1/2 and PI3K/Akt pathways [28, 29]. We therefore hypothesized the involvement of these two pathways in this process. Consistent with our hypothesis, TSC-Exos can induce rapid phosphorylation of AKT and ERK, which was later found to be attenuated by inhibitors LY294002 and PD98059. This suggests that the pro-survival signals in tenocytes can be rapidly activated by TSC-Exos to enhance cell viability. Here, we also found that TSC-Exos-induced proliferation and migration were reduced owing to these two inhibitors. In summary, these findings suggest that the PI3K/AKT and MAPK/ERK1/2 signaling pathways may play an important role in the proliferation and migration of tenocytes.

Next, we studied the role of TSC-Exos in regulating the inflammatory response in vivo. The early inflammatory response plays an important role in the tendon injury process which directly affects later healing of tendon tissue [30, 31]. In many types of tissue damage, pro-inflammatory macrophages (M1) can cause ECM degradation, inflammation, and cell apoptosis [32–34]. However, anti-inflammatory macrophages (M2) tend to regulate ECM balance and tissue healing [35]. Manning et al. showed that the transfer from pro-inflammatory M1 macrophages to anti-inflammatory M2 macrophages can enhance tendon healing [36]. Here, at 1 week after tendon injury, we found that, compared with that in the other groups, the proportion of CCR7+M1 macrophages in the TSC-Exos group was significantly downregulated, while the proportion of CD163+M2 cells was significantly upregulated. In addition, the expression of the M2 stimulating factor IL-10 was found to be increased, while the level of the inflammatory cytokine IL-6 was decreased. On the basis of these findings, we propose that TSC-Exos promotes anti-inflammation and tendon injury healing by modulating macrophages as well as associated cytokines. Cox-2, another pro-inflammatory factor, is closely related to fibrosis and adhesion after tendon injury [37]. In the present study, expression of cox-2 in the treatment group decreased significantly after tendon injury. This suggests that TSC-Exos may play an important role in improving tendon injury repair and remodeling.

Based on the H&E staining, we found that TSC-Exos treatment could result in a better arrangement of collagen fibers, with fibers more along the longitudinal axis of the tendon. This indicates that TSC-Exos has a positive effect on tendon injury repair, which is further demonstrated by studying changes in extracellular matrix-related factors during tendon repair. Collagen I is a major component of normal tendon collagen and is involved in tendon structure formation and its mechanical properties [38].

Type III collagen accounts for approximately 10% of the total tendon collagen; however, increased expression of type III collagen relative to type I collagen can inhibit the growth of collagen fibers, resulting in scar formation [39, 40]. A similar study has shown that an optimal ratio of type I/III collagen may be the main factor promoting high-quality healing of tendon injury [41]. In addition, the biomechanics of tendon tissue after scar formation decreases significantly, and large diameter collagen fibers are replaced by relatively small ones [42]. Reports show that biomechanical properties of tendon tissue are closely related to the diameter of tendon collagen fibers, and repaired tendon tissue with larger collagen fibrils is mechanically stronger than that with normal collagen fibrils [20, 43]. From the experimental results, we demonstrate that the ratio of type I/III collagen and the proportion of large-diameter fibrils in the experimental group were significantly higher compared to those of the other two groups, indicating that treatment with TSC-Exos might help to regulate the relative levels of ECM components and strengthen biomechanical properties for tendon injury healing.

Matrix metalloproteinases are a large family of zinc-dependent endopeptidases that are involved in the degradation and remodeling of ECM [4, 44]. TIMP is a natural inhibitor of the metalloproteinase family and can inhibit MMP degradation of ECM. Reports have shown that the balanced expression of MMPs/TIMPs plays an important role in ECM remodeling [45, 46]. In the present study, we found that, in the TSCs-Exos group, MMP-9 members of the MMP family were downregulated and inhibitor TIMP-1 was upregulated at 2 and 8 weeks. The results indicate that TSCs-Exo treatment balanced tendon ECM synthesis and degradation through modulating the metabolic balance between MMP-9 and TIMP-1. Furthermore, we also detected the expression of α-SMA and fibronectin, both of which showed a downward trend. Reports have shown that overexpression inhibition of α-SMA and fibronectin could restrain scar and fibrosis formation after tendon injury [4, 47]. Based on the above, TSC-Exos play an important role in inhibiting scar formation and promoting the high-quality healing of tendon injury.

Previous studies have shown that inhibition of cell apoptosis can promote tendon injury healing [48–50]. Indeed, the degree of cell apoptosis is closely related to the fiber structure and tissue repair. Therefore, reducing cell apoptosis in damaged tendon tissues would ultimately contribute to the recovery of histological and mechanical properties. In our experiments, we found that the number of apoptotic cells was markedly increased at week 2, compared to week 1. However, at either week 1 or 2, the extent of cell apoptosis in the TSC-Exos group was significantly reduced. These results indicate that a large number of apoptotic cells accumulate at the tendon repair site in the early stages of tendon healing, which can be reduced by TSC-Exos treatment, thus promoting injury healing.

In recent years, GelMA applied in the experiment has attracted extensive attention for tissue engineering applications due to its excellent biological performance and easily controllable photo-crosslinking properties [51, 52]. In addition to controlling shapes for personalized repair of soft tissue defects, its 3D structure is also suitable for cell growth and function [53]. To the best of our knowledge, the present study is the first to perform combined application of TSC-Exos and GelMA and it proved to be an effective integrated repair method in a rat model of Achilles tendon injury. This could be crosslinked to form the stable gel state in the window defect of Achilles tendon injury after the photo-crosslinking of GelMA, and then the gel is gradually absorbed in the body. Furthermore, mixing TSC-Exos in GelMA is easily delivered locally and prevents loss of exosomes. Thus, this method holds great potential for repairing tendon injuries in the clinic.

However, there are still several limitations to this study. First, after TSC-Exos stimulation, we did not set up multiple time points (such as 15, 30, and 60 min) to detect the phosphorylation of AKT and ERK in tenocytes, because only 30 min may not be the best time for the detection. Furthermore, the PI3K/AKT and MAPK/ERK1/2 signaling pathways may not be only pathways for TSC-Exos-related therapeutic effects, and other pathways (such as the BMP/Smad pathways, known to be pivotal in tendon healing) require further study and verification in the future. Second, measuring the collagen fiber diameter only indirectly assesses the strength of the injured Achilles tendon, which needs to be further determined by Biomechanical testing. Third, as exosomes contains a variety of proteins, mRNA, and miRNA, further identification and studies are needed to determine the specific substances in the exosomes associated with positive therapeutic effects.

## Conclusions

Taken together, the present study demonstrated that TSC-Exos obviously promotes the proliferation and migration of tenocytes in a dose-dependent manner, and this process may depend on the activation of PI3K/AKT and MAPK/ERK1/2 signaling molecules. On the other hand, TSC-Exos promotes high-quality healing of tendon injury by inhibiting inflammation, accumulation of apoptotic cells, and scar formation. These findings provide evidence for potential clinical application of TSC-Exos in tendon injury repair.

## Data Availability

The data that support the findings of this study are available from the corresponding author upon reasonable request.

## Abbreviations

TSCs: Tendon stem cells
TSC-Exos: TSC-derived exosomes
ECM: Extracellular matrix
TEM: Transmission electron microscopy
H&E: Hematoxylin-eosin
GelMA: Gelatin methacryloyl
CCR7: C-C chemokine receptor type 7
IL-10: Interleukin-10
IL-6: Interleukin-6
MMP-9: Matrix metalloproteinases-9
Timp-1: Tissue inhibitor of the metalloproteinase
COL1a: Collagen type I
COL3a1: Collagen type III
α-SMA: Alpha-smooth muscle actin
